# Sex Hormone Supplementation and Cardiovascular Disease Risk

**DOI:** 10.3390/medicina62010134

**Published:** 2026-01-09

**Authors:** Zofia Kampka, Magdalena Balwierz-Podgórna, Maciej T. Wybraniec

**Affiliations:** 1 Department of Cardiology and Structural Heart Diseases, Medical University of Silesia, 47 Ziołowa St., 40-635 Katowice, Poland; 2 Upper-Silesian Medical Center, 47 Ziołowa St., 40-635 Katowice, Poland; 3 First Department of Cardiology, School of Medicine in Katowice, Medical University of Silesia, 47 Ziołowa St., 40-635 Katowice, Poland; 4 European Reference Network on Heart Diseases—ERN GUARD-HEART, 1105 AZ Amsterdam, The Netherlands

**Keywords:** sex hormones, testosterone, hypogonadism, cardiovascular risk, cardiovascular disease

## Abstract

*Background and Objectives:* The fact that men are at a higher risk of cardiovascular disease (CVD) compared to women, regardless of concomitant risk factors, draws attention to the potential role of sex hormones in cardiovascular health. Hormonal therapies undoubtedly play a crucial role in reproductive and endocrine health; however, their cardiovascular implications remain complex and incompletely understood. This review aims at providing an updated overview of recent studies on this topic, highlighting the practical clinical aspects and knowledge gaps. *Materials and Methods:* This review synthesizes recent clinical studies regarding the cardiovascular impact of female hormone replacement therapy (HRT) and testosterone replacement therapy (TRT). *Results:* It seems that both hormonal deficiency and excess can exert detrimental effects on the cardiovascular system. While HRT and TRT offer benefits to specific patient populations, their broad biological actions can lead to adverse effects. This creates a sophisticated and delicate relationship between hormonal balance and heart health, complicating the determination of universal safety profiles and use indications. *Conclusions:* The risk–benefit ratio of hormonal therapies remains a critical concern in clinical practice. Because cardiovascular effects vary significantly based on individual patient profiles, a nuanced approach to prescribing is necessary. Further research is required to bridge knowledge gaps and refine safety guidelines for the use of sex hormones in cardiovascular prevention and treatment.

## 1. Key Points

1. Low testosterone levels seem to be associated with increased cardiovascular (CV) risk and mortality; however, the relationship remains complex. It is still debatable whether testosterone should be perceived as a biomarker of overall health or an independent cardiovascular risk factor.
2. It seems that clinically tailored transdermal TRT is not associated with increased incidence of major cardiovascular events and mortality; moreover, it may even be protective when testosterone levels are normalized.
3. While TRT showed promising results in metabolic syndrome treatment, due to inconsistent study results and possible conflicts of interest, further studies are needed.
4. The impact of hormone replacement therapy (HRT) on cardiovascular diseases (CVD) in women remains inconclusive. Evidence suggests that initiating HRT within 10 years of menopause or before age 60 may lower CVD risk and mortality compared to later initiation.
5. Transdermal HRT is considered safer than oral therapy—particularly in women with obesity or dyslipidemia—because it carries a lower risk of venous thromboembolism (VTE) and more favorable effects on lipid metabolism.

## 2. Introduction

The gender disparity in cardiovascular disease (CVD) prevalence is a subject of research, with sex hormones playing a pivotal role [1]. Even after adjusting for the influence of comorbid risk factors, such as dyslipidemia, diabetes, or cigarette smoking, men are twice as likely to be affected by CVD compared to women [2]. Sex hormones in women have a profound impact on phenotype, lipid and calcium metabolism, coagulation processes, protein synthesis, and bone density. These hormones are equally crucial for men, as deficiencies can lead to infertility [1]. Estrogens and progestogens are used in several clinical settings, including hormone replacement therapy (HRT), hormonal contraception, and the treatment of hormone deficiencies. Despite their therapeutic benefits, the administration of sex hormones may also lead to adverse effects due to their broad range of biological actions, which depend on factors such as sex, age, and dosage.

The role of testosterone in CVD risk is equivocal, with both excess and deficiency being potentially harmful [1]. The risk–benefit ratio of testosterone replacement therapy (TRT) is still a subject of debate due to CV safety concerns [3]. The aim of this review is to present the most up-to-date findings, stressing the clinically useful aspects, knowledge gaps, and current evidence limitations.

## 3. Hypogonadism

**Figure 1:**
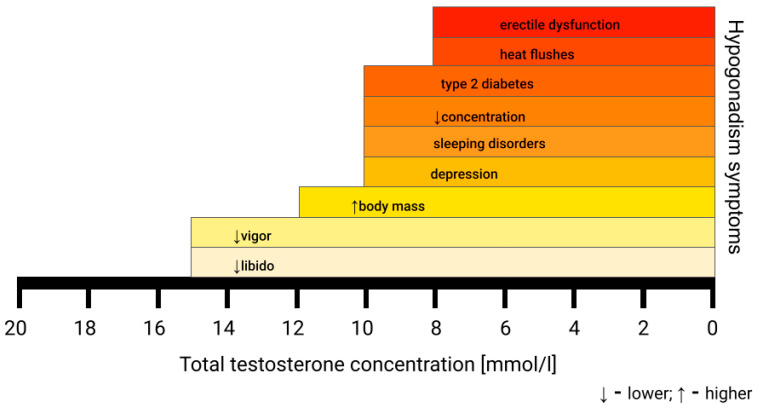
Symptoms of hypogonadism based on the serum total testosterone concentration. The first signs of hypogonadism can be subtle and non-specific [4].

The term “hypogonadism”, according to the European Association of Urology, means a low level of serum testosterone, negatively influencing quality of life and organ functions, which is a combined clinical and biochemical syndrome. Although the number of men diagnosed with hypogonadism is growing, it still remains underdiagnosed. Hypogonadism can be caused by multiple underlying conditions, which can be classified as genetic (Klinefelter syndrome, Kallmann syndrome), endocrinological (pituitary, thyroid or testicular disorders), metabolic (obesity, diabetes mellitus), inflammatory (chronic inflammatory bowel diseases, chronic obstructive pulmonary disease, rheumatoid arthritis), psychiatric (depression, anorexia nervosa) and other (hemochromatosis, vitamin D deficiency, HIV infection). It can also be of iatrogenic (radiotherapy, pharmacotherapy of glucocorticoids, opioids, or antipsychotic drugs) or iatrogenic background. Late-onset hypogonadism (LOH) is caused by irreversible hypothalamic–pituitary–gonadal axis impairment [4]. It results in a gradual reduction in testosterone levels, approximately at a rate of 1–2% per year, causing symptoms suggestive of androgen deficiency, affecting 20% men over 60 years and 50% over 80 years [3,5]. Patients may complain not only of sexual-related symptoms, such as low libido and erectile dysfunction, but also of non-sexual ones [4]. These are non-specific and encompass, i.a., tiredness, cognitive impairment, reduced muscle strength, depression, anemia, obesity, body hair loss, hot flushes, sleep disorders, diabetes, sarcopenia, osteopenia, or osteoporosis [3,4,6]. The severity of symptoms is dependent on the total testosterone level, as presented in Figure 1. It seems that hypogonadism and its comorbidities are closely tied, creating a relationship resembling a vicious circle.

A total serum testosterone concentration under 12.1 nmol/L is the lower limit of the normal testosterone level according to the European Association of Urology (EAU) Guidelines. For an accurate diagnosis of hypogonadism, samples must be collected between 7:00 and 11:00 a.m. in a fasting state and should be deferred during acute illness. The results must be confirmed in two or three samples taken on separate days [3,4,7]. Laboratory test results with the presence of at least one symptom of hypogonadism are sufficient to consider TRT. However, the lower threshold of the total testosterone serum concentration remains a subject of debate, ranging from 11 nmol/L to 15 nmol/L [4]. These differences are a result of symptom-specific thresholds, with hot flushes and erectile dysfunction being present at a total serum testosterone concentration under 8 nmol/L and more subtle ones, like fatigue and lower libido, appearing below 15 nmol/L [3,4]. This causes inconsistency between guidelines from different associations. The lack of commonly accepted age-related reference ranges further complicates making the diagnosis [3]. Moreover, serum total testosterone is usually measured by radioimmunoassay and immunometric assays, which may cause discrepancies in results between laboratories due to differences in the antibodies used. The gold standard measurement is liquid chromatography tandem mass spectrometry; however, it is not widely available [3].

When the total testosterone serum concentration does not match the clinical symptoms or is within the lower limit (8–12 nmol/L), the diagnostic process should incorporate sex hormone-binding globulin (SHBG) concentration in order to calculate the free testosterone level, which is biologically active [2,4]. The direct free testosterone level measurement is challenging and not widely available [3,4]. Notably, some conditions may be related to reduced SHBG levels, such as insulin resistance, diabetes mellitus, obesity, hypothyroidism, and pharmacotherapy (glucocorticoids, anabolic androgenic steroids) [2,3]. The lower limit of free testosterone remains controversial and ranges from 170 to 310 pmol/L, with concentrations below 225 pmol/L or 243 pmol/L being suggested as cut-off values by recent research. Another suggested approach is to base clinical decisions on the normal range given by the laboratory. Free testosterone concentration should not be used in TRT monitoring due to a paucity of studies in this field [3,4].

Several modalities of testosterone administration exist—oral (currently not recommended due to low bioavailability or adverse effects), trans- and subdermal, intramuscular, buccal (not available in Europe), and nasal. The transdermal route is widely used due to its safe and non-invasive character [8,9]. While hypogonadism in young men caused by hypothalamic–pituitary or testicular disorders belongs to the basic indications for TRT, pharmacotherapy in elderly men remains a subject of debate. According to up-to-date research, TRT offers significant benefits despite potential risks [3]. TRT is found to improve overall quality of life by influencing cognitive function, mood, body composition, and sexual satisfaction [8].

## 4. Testosterone Replacement Therapy and Cardiovascular Risk

**Figure 2:**
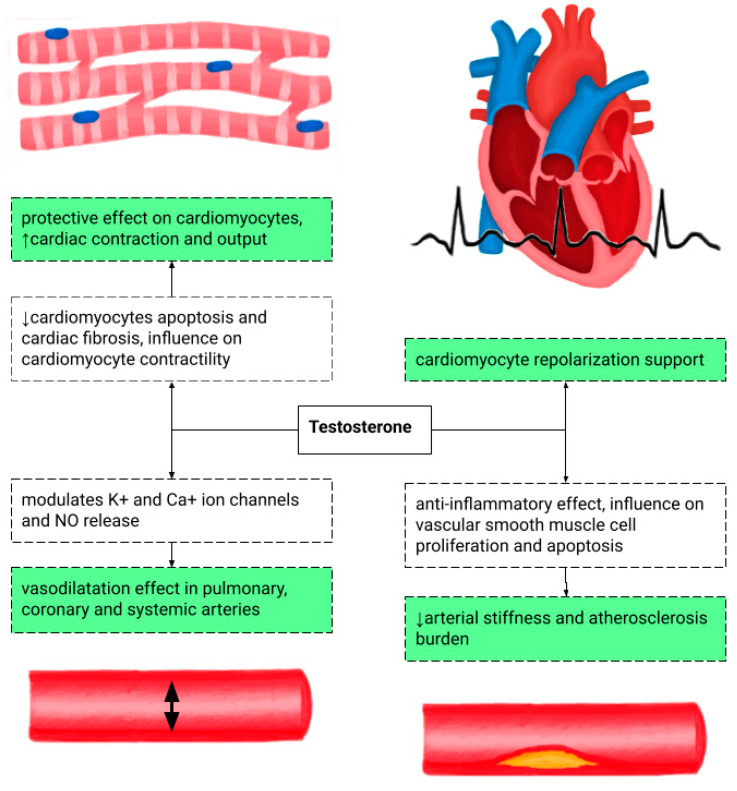
The potentially positive influence of testosterone on the cardiovascular system at the cellular and tissue levels [11,12]. NO—nitrous oxide.

The influence of testosterone on the heart is presented in Figure 2. The recommendations for TRT based on age and testosterone concentration are shown in Table 1. TRT in hypogonadal patients reduces pro-inflammatory cytokine levels and endothelin-1, a vasoconstrictor peptide, therefore modulating CAD development. This is one of the possible explanations of delayed ST-segment depression on a treadmill stress test in patients with TRT. It also lowers the risk of torsade de pointes by shortening the QT interval [8,9,10].

Notably, patients without TRT and those in the subtherapeutic range of testosterone are at a higher risk of stroke, myocardial infarction, and death [8]. A retrospective study by Sharma et al. investigated the role of TRT, encompassing injection, gel, or patch administration routes, in MI, stroke, and death risk reduction. The normalization of the testosterone level in men without a history of CV disease was associated with lower all-cause mortality, MI, and stroke risk. However, patients with TRT below the therapeutic range did not experience the aforementioned benefits. The exact pathophysiological pathway behind these results remains poorly understood [14]. Conversely, testosterone may potentially heighten CV risk by increasing platelet aggregation and lowering HDL levels [14]. TRT may also result in polycythemia and increased blood viscosity due to erythrocyte proliferation. It is recommended to monitor hemoglobin levels and discontinue TRT when hematocrit exceeds 54% [8]. However, according to research by Sharma et al., no association was found between TRT in men with low or moderate risk of venous thromboembolism and the occurrence of deep vein thrombosis and pulmonary embolism [15].

The CV risk of TRT was assessed in the TRAVERSE study, which was a multicenter, randomized, double-blind, placebo-controlled, noninferiority trial that enrolled 5246 participants and observed them up to 5 years. All men had high CV risk or pre-existing CV disease. The patients were randomly administered 1.62% testosterone gel or a placebo gel, and the mean treatment duration was 22 months. No significant difference was found between the TRT and placebo in terms of major adverse cardiovascular event (MACE) incidence, encompassing CV mortality, nonfatal myocardial infarction, or nonfatal stroke. Interestingly, the TRT group had a higher incidence of nonfatal arrhythmias requiring intervention, atrial fibrillation, acute kidney injury, and pulmonary embolism, especially in men with thrombophilia [8,16].

Complementing these findings, a study by Baillargeon et al. investigated the association between intramuscular testosterone administration in TRT and myocardial infarction (MI) in a retrospective study. TRT did not increase the risk of MI; moreover, the treatment in men at high risk of MI demonstrated a modest protective effect against it [17]. In the research by Wallis et al., which assessed the results of cumulative testosterone dose exposure, an inverse association was found between TRT exposure, CV events, and mortality. A longer duration of TRT with a median of 35 months was associated with a lower risk of cardiovascular events, prostate cancer, and death compared to controls [5].

Opposite results were reported by the Testosterone in Older Men with Mobility Limitations (TOM) trial, which enrolled 209 men with a mean age of 74 years and limitations in mobility. They were randomly assigned to receive a testosterone or placebo gel for six months. While in the testosterone group, an improvement in muscle strength was observed, the risk of cardiovascular-related adverse events was also higher compared to the placebo group. The study population was unique, relatively small, and had high rates of comorbidities, such as chronic conditions, including pre-existing heart disease, diabetes, and hypertension. While the results of the TOM trial raise concerns regarding the safety of TRT, the generalizability of those findings is limited [18].

Another study worth mentioning is a meta-analysis by Xu et al., which included 2994 middle-aged or elderly men with low testosterone levels and/or chronic diseases. The results revealed that TRT was associated with an increased risk of adverse cardiovascular events. Moreover, the outcomes of particular studies included in the meta-analysis varied, depending on the source of funding, with trials sponsored by the pharmaceutical industry showing a lower risk of cardiovascular-related events on TRT [19]. These discrepancies underscore the need for a critical appraisal of industry-funded research.

All in all, potential benefits and hazards should be carefully considered before implementing TRT in middle-aged and elderly men; however, taking novel studies into consideration, it does not seem to affect the CV risk in a negative way [16]. Still, more research, independent of the source of funding, is needed to corroborate it. All the most vital studies on TRT and CV risk or mortality are summarized in Table 2.

## 5. Testosterone Replacement Therapy and Mortality

A low testosterone level is linked to increased mortality, which has been proven in two comprehensive meta-analyses. One meta-analysis, encompassing 43,041 men—mean age of 63.5 years—by Corona et al., showed that a low testosterone level is associated with a higher risk of overall and cardiovascular-related mortality. This relationship is particularly visible in younger men, while the CV risk in elderly men depends more on comorbidities than solely on a low testosterone level [20]. Similar results were obtained in a previous meta-analysis by the same author, where lower testosterone and higher 17-β estradiol levels were linked to increased cardiovascular disease and cardiovascular mortality risk. Interestingly, no such association between dehydroepiandrosterone (DHEAS) and cardiovascular disease has been observed [1].

Apart from research papers mentioned in the section “Testosterone Replacement Therapy and Cardiovascular Risk”, which showed an inverse association between TRT and mortality, it is pertinent to note a retrospective study by Shores et al., which included 1031 middle-aged and elderly hypogonadal men with a high comorbidity burden (a mean of 6.7 conditions). TRT was associated with reduced mortality (10.3% in the TRT group compared with 20.7% in the control group), especially in younger men (below 60 years) and in patients burdened with diabetes or CAD [6].

A study by Gencer et al. shed light on the relationship between mortality and total testosterone levels in patients with acute coronary syndrome. While a low testosterone level was linked to a worse lipid profile and elevated levels of high-sensitivity C-reactive protein, high-sensitivity troponin T, and N-terminal-proB-type natriuretic peptide, no significant association was found between a low total testosterone level and the mortality rate after adjusting for potential confounders. Notably, in the group of men aged > 65 years, the mortality was higher, and the age group represented the lowest testosterone tertile in the study [21]. The results obtained in this specific population of patients raise the question of whether the testosterone level serves as a surrogate biomarker of general health or is a causal risk factor for mortality. However, when synthesizing these results with broader research, TRT rather seems to be beneficial in terms of mortality, especially in men < 60 years with CV disease.

The relationship between the testosterone level and mortality is complex, as a low testosterone level may be viewed as a marker of poor general health conditions, especially in elderly men. In this light, TRT does not appear to be a causal treatment. Moreover, the existing research suggested promising results, predominantly in middle-aged men. Therefore, TRT in elderly men should be carefully considered, thinking about the hazard–benefit ratio.

## 6. Testosterone Replacement Therapy and Metabolic Disorders

TRT may also indirectly modulate CV risk by having an effect on the lipid profile, waist circumference, and glycemic control. TRT reduces the serum level of total cholesterol (TC), low-density lipoproteins (LDLs), and triglycerides (TGs), simultaneously increasing the high-density lipoprotein level (HDL). The maximal lipid-lowering influence can be observed after 6–12 months of TRT, with the first effects visible after 4 weeks. TRT promotes reductions in body mass and visceral adiposity, which can be causes of hypogonadism in men. Moreover, TRT is associated with reducing carotid intima-media thickness, a surrogate atherosclerosis marker, and improving insulin sensitivity, leading to better glycemic control and a decrease in hemoglobin A1c (HbA1c) [8,22,23]. TRT seems to have a positive effect in men with frailty syndrome by positively influencing body composition [1].

Interestingly, in a randomized study by Shigehara et al., hypogonadal Japanese men with metabolic syndrome were administered intramuscular testosterone injections. TRT lasting for 1 year proved to reduce waist circumference, body fat percentage, serum TG levels, fasting plasma glucose (FPG), and HbA1c. However, TRT showed no significant impact on parameters such as HbA1c, TC, HDL, FPG, and BP when compared to the control group. The possible differences in TRT influence on metabolic parameters may have been caused by the relatively small population included in a study by Shigehara. Secondly, it was a sub-analysis of the EARTH study, performed retrospectively [24].

Despite these promising findings, further prospective randomized studies are required to evaluate the long-term influence of TRT on the components of metabolic syndrome.

## 7. Testosterone Replacement Therapy and Heart Failure

A reduced testosterone level serves as an independent risk marker for heart failure (HF) and worse outcomes in patients of both sexes. Male patients with HF and low testosterone levels are at a higher risk of decreased exercise capacity and unfavorable prognosis. In a meta-analysis by Mustafa et al., testosterone supplementation in moderate to severe HF was associated with an increased 6 min walk test (6MWT) distance by approximately 54 m, which is a better result than that achieved by standard HF pharmacotherapy and cardiac resynchronization therapy. Moreover, the peak VO2 increase observed in patients with testosterone supplementation was higher than that observed in cardiac resynchronization therapy trials. The improved exercise capacity had a complex pathophysiological background, potentially encompassing peripheral vasodilatation, muscle mass increase, anti-inflammatory effect, and hemoglobin rise. The phenomenon was observed during treatment lasting from 12 to 52 weeks [22].

Another interesting viewpoint was provided by a study assessing the CV risk in men treated for prostate cancer. It was conducted on a population-based cohort of 25,436 Danish men and showed a twofold higher risk of heart failure and ischemic stroke in men receiving first-line palliative treatment for prostate cancer in comparison to cancer-free men. It is worth stressing that palliative treatment involved androgen deprivation therapy, which was administered to 94% in this group, chemotherapy, radiotherapy, or surgery. The endocrine treatment involved antiandrogens, gonadotropin-releasing hormone agonists, and orchidectomy. Such an association was not found in men with “curative-intent” treatment, encompassing radical prostatectomy and radio- and brachytherapy. Interestingly, the risk of myocardial infarction did not differ in any treatment group [25].

TRT also results in New York Heart Association (NYHA) class improvement, which is present in over one in three patients with HF [8]. However, no improvements in echocardiographic cardiac function measurements were observed [22]. The influence of testosterone on the CV system is complex, as it may also increase salt and water retention, potentially leading to edema, hypertension, and HF decompensation episodes [17]. A meta-analysis of randomized controlled trials by Tao et al. concluded that TRT within a physiological range in congestive HF patients was not significantly associated with improved exercise capacity, cardiac function, quality of life, or clinical outcome [26].

All in all, the research results remain inconclusive, with inconsistent results and a paucity of data in HpEF. More trials are needed to call TRT a promising option in the future for patients at risk of developing HF. In patients with established HF, steroid treatment and TRT should generally be avoided due to the risk of provoking fluid overload and HF decompensation.

## 8. Anabolic Steroids and Cardiomyopathy

It must be stressed that there is a substantial difference between TRT and anabolic androgenic steroid (AAS) misuse for muscle mass stimulation and physical performance improvement. AASs are associated with hypertension, dyslipidemia, and premature atherosclerosis. These, combined with abnormal platelet aggregation and greater erythropoiesis, result in a higher CV risk. According to a study by Windfeld-Mathiasen et al., performed on men in Danish fitness centers, AASs are associated with a higher risk of venous thromboembolism, acute myocardial infarction, arrhythmias, cardiomyopathy, and heart failure [27].

AASs promote left ventricle (LV) remodeling by activating the renin–angiotensin–aldosterone system (RAAS); however, higher myocardial thickness is not accompanied by increased contractility due to fibrosis and changes in matrix collagen deposition. This also influences electrical conduction, resulting in higher arrhythmogenicity and weaker contractility. It has been observed that, apart from decreasing LV systolic function, chronic AAS use also promotes fibrosis of the right ventricle (RV), RV diastolic dysfunction, and decreased RV strain in echocardiography. The diagnostic process of AAS-induced cardiomyopathy includes transthoracic echocardiography, cardiac magnetic resonance imaging, and coronary computed tomography angiography (CCTA) or coronary angiography to determine the ischemic background. While the presenting symptoms may range from dyspnea to ventricular tachycardia, the common feature of AAS-induced cardiomyopathy is LV dysfunction [28,29]. According to the existing literature, AASs can also be linked to cardiac death due to dilated cardiomyopathy, hypertrophic cardiomyopathy, and myocarditis, which were post-mortem findings in AAS users [30,31].

While TRT under clinical supervision seems to be a safe therapeutic option, in hypogonadal men who plan to reproduce, TRT is contraindicated due to fertility impairment via suppression of gonadotrophins, endogenous testosterone secretion, and spermatogenesis. It is recommended to start gonadotrophin treatment instead [7].

## 9. Hormonal Replacement Therapy in Women and Cardiovascular Disease Risk

HRT has been shown to be beneficial for postmenopausal women, particularly in reducing menopausal symptoms and protecting the skeletal system. However, scientific evidence regarding its effects on cardiovascular risk remains inconclusive, with some studies reporting either an increase or a decrease in risk depending on the type of hormones administered [32].

One-third of menopausal women suffer from the following symptoms: hot flashes, night sweats, muscle and limb pain, dyspareunia, increased susceptibility to urinary tract infection, and bone fractures, which are consequences of hormonal transformation. During menopause, the production of endogenous estrogen, progesterone, and testosterone decreases, while the levels of follicle-stimulating hormone (FSH) and luteinizing hormone (LH) increase [33]. The reduction in estrogen production is associated with a higher risk of metabolic syndrome features, diabetes (DM), and, subsequently, cardiovascular diseases (CVDs).

One of the largest and most important studies in this field was that resulting from the Women’s Health Initiative (WHI). In this trial, 27,347 postmenopausal women from 40 medical centers across the United States (USA) were analyzed with respect to the risk of developing coronary artery disease (CAD) and the risk of experiencing ischemic stroke. In the first part of the study, the participants were randomized into subgroups; the first subgroup took 0.625 mg of oral conjugated equine estrogens combined with 2.5 mg of medroxyprogesterone acetate, and the second subgroup took a placebo [34,35]. Combined hormone therapy was associated with increased risk of CAD (HR 1.24; CI 95%, 1.00–1.54), with a higher rate in the first year. Similar results were obtained for ischemic strokes with HR 1.44 (CI 95%, 1.09–1.90), regardless of age, presence of previous CVD, or history of ischemic stroke. The second part of the study included postmenopausal women after hysterectomy, who were randomized to receive 0.625 mg of oral conjugated equine estrogens or a placebo [36]. This hormonal therapy did not significantly affect the risk of CAD (HR 0.91; CI 95%, 0.75–1.12), but it was associated with an increased risk of ischemic stroke (HR 1.39; CI 95%, 1.10–1.77). The WHI trial has been subject to criticism due to methodological limitations and potential bias, which must be taken into account for an accurate interpretation of the results—particularly the inclusion of women aged 60 to 79 years.

However, many studies have been carried out since the WHI. A large-scale meta-analysis by Yang et al., including 26,166 postmenopausal women, indicated that combined hormonal therapy (estrogens and medroxyprogesterone acetate) did not influence coronary events, myocardial infarction (MI), stroke, cardiac death, total death, or revascularization. What is more, estrogen therapy alone, evaluated in 12,847 postmenopausal women, was also proven to have no effect on coronary events, MI, cardiac death, total death, and revascularization, but it was related to a 27% increased risk of stroke [37].

Nowadays, HRT is not recommended for CVD prevention, neither by the American College of Obstetricians and Gynecologists nor the Endocrine Society [38,39].

CVD risk reduction has been shown to vary depending on the age at which hormone replacement therapy (HRT) is initiated in postmenopausal women. A randomized controlled trial by J. Rossouwe et al. showed a tendency toward a greater reduction in CVD risk among patients who began HRT within 4 years of menopause compared with those who started more than 10 years after menopause [40]. Similar results were obtained in a 2015 meta-analysis for both combined HRT and estrogen-only therapy, in the context of not only primary but also secondary prevention. Women who initiated HRT within 10 years after menopause presented a lower incidence of mortality and CVD compared with placebo subgroups. However, the first subgroup also demonstrated an increased risk of venous thrombosis (VTE), with no observed impact on stroke compared with a placebo. In contrast, patients who began HRT more than 10 years after menopause exhibited an increased risk of stroke and no significant effect on mortality or CVD incidence [41].

Estrogen deficiency is associated with endothelial dysfunction through a reduced vasodilatory effect, decreased inhibition of sympathetic activity, and impaired nitric oxide (NO) synthesis [42]. The 2016 ELITE (Early Versus Late Intervention Trial With Estradiol) study showed that a HRT with oral estrogens combined with vaginal progestogens subgroup was associated with a slower progression of subclinical arteriosclerosis, as measured by the rate of change in carotid artery intima-media thickness (CIMT) among women within 6 years after menopause compared with a placebo subgroup. Furthermore, no such effect was noted in women more than 10 years after menopause [43].

The studies described above focused on the influence of oral HRT on CVD risk. Nevertheless, transdermal therapy has also been evaluated because of its different metabolic profile and the potential for lower, yet equally effective doses in alleviating menopausal symptoms compared with oral administration. No randomized controlled trials have assessed the impact of the route of estrogen administration on CVD risk. Clinical studies have shown a tendency toward a higher risk of VTE with systemic HRT compared with transdermal therapy [44,45]. Similar findings were reported among patients carrying the most common prothrombotic mutations, such as factor V Leiden and prothrombin G20210A [46]. What is more, clinical research has indicated a relationship between HRT and lipid metabolism. HRT has been shown to reduce LDL cholesterol levels regardless of the route of administration [47]. Additionally, oral HRT has been associated with increased levels of high-density cholesterol (HDL) and triglycerides. In contrast, transdermal HRT did not show a significant effect on HDL levels, but it was associated with a significant reduction in triglyceride concentrations [48].

To summarize, specific recommendations have been developed for the use of HRT, taking cardiovascular risk into account. HRT use is considered safe in low-risk patients—women under 60 years of age, within 10 years of menopause onset, with an estimated 10-year risk of atherosclerotic cardiovascular disease (ASCVD) below 5%, and without an increased risk of breast cancer or a history of VTE. The intermediate-risk subgroup includes women with at least one chronic condition, an estimated ASCVD risk of 5–10%, and elevated risk for breast cancer. The most optimal and safest route of administration for women with obesity or dyslipidemia is transdermal HRT [48,49]. HRT has been shown to increase blood pressure [50]. Caution is recommended when initiating the therapy in women with arterial hypertension. In uncontrolled blood pressure with values above 180/110 mmHg, HRT initiation should be delayed until normalization is achieved. In women with DM, HRT has been associated with improved glycemic control and a positive effect on insulin resistance [51]. Moreover, the use of HRT has been linked to a reduced risk of new-onset DM [52]. In high-risk populations, systemic HRT is generally contraindicated, particularly among patients with an estimated ASCVD risk greater than 10%, congenital heart disease, or breast cancer. HRT should also be avoided in cases of acute cardiovascular disease, VTE, stroke or transient ischemic attack (TIA), and pulmonary embolism (PE) due to the potential for exacerbating these conditions [33]. An overview of key studies evaluating the effects of HRT on cardiovascular risk factors and cardiovascular events in postmenopausal women is presented in Table 3.

To summarize, evidence indicates that the cardiovascular effects of HRT depend strongly on hormone type, administration route, timing of initiation, and individual patient risk factors. Initiating HRT within 10 years of menopause may slow subclinical atherosclerosis and have a neutral or slightly beneficial effect on cardiovascular outcomes, especially with transdermal or standard-dose oral estrogens, whereas late initiation or combined systemic therapy in older women is associated with higher risks of stroke and venous thromboembolism. Consequently, HRT is not recommended for the prevention of cardiovascular disease, and its use should focus on symptom relief, taking age, comorbidities, and route of administration into account. These conclusions are supported by large RCTs, meta-analyses, and current clinical guidelines.

## 10. Gender-Affirming Hormone Therapy in Transgender Women and Cardiovascular Disease Risk

Gender-affirming hormone therapy (GAHT) has become an important component of the gender transition process. The estimated prevalence of transgender individuals is up to 2% of adults in the USA [53]. Transgender women typically receive systemic or transdermal estrogen therapy, often administered with a gonadotropin-releasing hormone analogue or an antiandrogen agent. Furthermore, antiandrogens, such as spironolactone or finasteride, are commonly used to inhibit the physiological effects of testosterone [54]. The primary goal of the treatment is to achieve and maintain physiologic levels of estrogen to promote the development of female secondary sex characteristics while suppressing endogenous androgens to minimize or reverse male secondary sex characteristics. Formulations available in the treatment process are oral and transdermal preparations, administered daily, as well as intramuscular pharmaceuticals used once a week or less frequently. Beyond the physical changes, GAHT is recognized as a long-term medical intervention that may influence multiple physiological systems, including lipid metabolism, insulin sensitivity, vascular function, and coagulation pathways [55].

Transgender women, reported as male-to-female, have been noted to have approximately twice the risk of CVD than cisgender women. No randomized control trials have been conducted to date; therefore, available data are limited to clinical trials and observational studies. Data from the Behavioral Risk Factor Surveillance System collected between 2014 and 2017 showed that transgender women demonstrated a higher likelihood of MI compared with cisgender women (OR 2.56; 95% CI, 1.78–3.68; *p* < 0.01). However, no such correlation was observed between male-to-female entities and cisgender men, even after adjustment for demographic factors, comorbidities, and physical activity using logistic regression models. Nonetheless, the authors of the study also emphasized the potential influence of other factors, such as socioeconomic disparities, increased stress levels, social abuse, and psychoactive substance use, which may lead to systemic inflammation and, consequently, increased CVD risk. Evidence from large population-based studies indicates that conventional cardiovascular risk factors, such as smoking, overweight and obesity, hypertension, and diabetes, occur more frequently in transgender individuals than in cisgender comparison groups. These factors may lead to systemic inflammation and, consequently, increased CVD risk [56].

Transgender women have been reported to present higher overall mortality than the general population, as assessed by the standardized mortality ratio (SMR). Among various death causes, cardiovascular reasons were more frequent than expected (OR 1.64; 95% CI, 1.43–1.87). However, the true magnitude of risk is challenging to determine due to historical differences in hormone regimens, limited monitoring protocols, and the inclusion of older estrogen formulations no longer routinely prescribed. These factors highlight the need for caution when extrapolating earlier mortality data to contemporary GAHT practices [57].

However, a comparison between male-to-female transforming patients receiving GAHT and transgender women without such treatment showed a beneficial effect of estrogen on the cardiovascular system by lowering HDL levels and CIMT in a Thai population. The study focused on CVD risk factors assessed through laboratory test results, CIMT measurements, and physical examination findings, but it did not evaluate major adverse cardiovascular events (MACEs), limiting its ability to determine the overall impact of GAHT [58].

From a clinical standpoint, the existing evidence emphasizes the need for personalized cardiovascular risk assessment both before starting GAHT and throughout long-term follow-up. This approach should include regular monitoring of blood pressure, lipid levels, glucose metabolism, and body composition, alongside active management of modifiable risk factors. For individuals with elevated baseline cardiovascular risk, transdermal estrogen may be preferable due to its more favorable metabolic and thrombotic profile compared with oral formulations. Nevertheless, firm clinical guidelines are limited by the lack of randomized controlled trials assessing hard cardiovascular outcomes.

## 11. Contraception and Cardiovascular Disease Risk

Endogenous estrogens exert a protective effect against cardiovascular diseases in premenopausal women. However, this cardioprotective effect diminishes after menopause and becomes comparable to that observed in males [59]. Estrogens influence lipid metabolism, glucose regulation, vascular responsiveness, and blood pressure, contributing to the reduced risk of coronary artery disease, ischemic heart disease, aortic stenosis, VTE, atrial fibrillation, DM, and hyperlipidemia [60]. Although endogenous estrogens inhibit the proliferation and migration of vascular smooth muscle cells, thereby preventing the formation of atherosclerotic plaques, exogenous hormones are related to increased risk of arterial hypertension, as well as the development of arterial and VTE, due to the enhanced production of coagulation factors [61].

Endogenous progestagens demonstrate beneficial effects on the cardiovascular system, expressed as vasodilatation and hypotensive effects. In animal models, higher progestagen levels were related to better protection from coronary artery hyperactivity, decreased platelet aggregation, and oxidative stress markers [62].

### 11.1. Combined Oral Contraception and Cardiovascular Risk

Combined oral contraception (COC) with estrogen and progestagen components was the most commonly prescribed contraceptive method in the USA, used by about 11% of 15–44-year-old women in 2022–2023 [63].

The estrogen component is associated with a fourfold increased risk of VTE, occurring more frequently in individuals with obesity, and up to a sevenfold increase in those with particular thrombophilia types (e.g., factor V Leiden G1691A mutation, prothrombin G20210A mutation) [64]. An increased relative risk of VTE associated with COC use was linked to higher doses of the estrogen component (RR 1.20, 95% CI 0.85–1.71 for 50 μg compared to the reference dose of 30–40 μg). What is more, the risk ratio of VTE decreased with the duration time of COC use (<1 year RR 4.17, 95% CI 3.73–4.66; 1–4 year RR 2.98, 95% CI 2.73–3.26, and >4 year RR 2.76, 95% CI 2.53–3.02; *p* < 0.001) [65]. The meta-analysis by Oedingen et al. showed that all COCs included in the study were associated with an increased absolute risk of VTE. However, the use of COCs containing the lowest possible dose of ethinylestradiol (low dose—20 μg or moderate dose—30–40 μg) combined with levonorgestrel was associated with a minimized risk. Concerning the progestagen component, the lowest relative risk was obtained for levonorgestrel with 30–40 μg of estrogens compared to formulations with desogestrel (RR: 1.46; 95% CI: 1.33–1.59), drospirenone (RR 1.40 95%; CI: 1.26–1.56), gestodene (RR: 1.27; 95% CI: 1.15–1.41), and cyproterone (RR: 1.29; 95% CI: 1.12–1.49) [66].

The estrogen component of COCs has also been associated with elevated blood pressure in women with pre-existing arterial hypertension, as well as with the development of the condition in previously normotensive women by stimulation of the renin–angiotensin–aldosterone system (RAAS), antidiuretic hormone secretion, and enhanced sympathetic nervous system activity [67]. The risk increased with the duration of therapy, with a higher risk observed after 1–2 years of use (RR 1.22; 95% CI, 0.75–1.99) and an even greater risk after more than two years (RR 1.96; 95% CI, 1.03–3.73) [68]. COCs were related to an increased risk of elevated blood pressure in low (<30 μg) and moderate (30–50 μg) doses of ethinyl estradiol, but there are limited data considering dose dependency. A small study demonstrated that reducing high estrogen doses (50–100 μg) to a lower dose (30 μg) was associated with a significant decrease in blood pressure levels, although not to pre-treatment values, suggesting a possible dose–response relationship [69]. However, high doses >50 μg have not been recommended since the 1970s [67].

Another composite of cumulative cardiovascular risk is the increased incidence of MI (RR 1.6; 95% CI 1.2–2.1) and ischemic stroke (RR 1.7; 95% CI 1.5–1.9) associated with COC use. No significant differences were observed with respect to the generation of progestagens, but the risk of both adverse events appeared to be dose-dependent on the estrogen component [70]. Notably, an especially high risk of MI was presented by smoking and hypertensive individuals (RR 19.0; 95% CI = 4.7–7.8) [71]. However, the relative risk of major coronary artery disease, ischemic stroke, and cardiovascular death seemed to be reversible after discontinuation of the therapy and amounted to 0.8 (95% CI: 0.6–1.0), 1.0 (95% CI: 0.7–1.3), and 0.9 (95% CI: 0.7–1.2), respectively [72]. These results may be consistent with a recent large cohort study, which demonstrated that the use of oral hormonal contraception was not associated with increased risk and instead showed a statistically significant reduction in the risk of cardiovascular disease events (HR 0.91; 95% CI: 0.87–0.96), coronary heart disease (HR 0.88; 95% CI: 0.81–0.95), heart failure (HF) (HR 0.87; 95% CI: 0.76–0.99), and atrial fibrillation (HR 0.92; 95% CI: 0.84–0.99). However, the study referred to the use of any type of oral hormonal contraception and did not distinguish between current use and past use. Additionally, the questionnaire format may have introduced potential bias [73].

Hormonal oral contraception was related to a lower risk of type 2 DM (T2DM) development (HR 0.93; 95% CI: 0.8–0.98) in a recent population-based study [74]. Furthermore, a favorable effect of hormonal contraception was observed in individuals with type 1 DM (T1DM), as evidenced by reduced progression of coronary artery calcification evaluated with electron beam computed tomography (16.5% vs. 35.5% in controls, who had never used hormonal birth control). The study mostly considered oral hormonal contraception, without differentiation between the specific types [75].

Estrogen’s component in COCs was also associated with significant changes in lipid profiles. In a cohort of 828 adolescent females over an average follow-up period of 22 months, individuals who began using oral contraceptive administration showed significantly greater increases in LDL cholesterol (15.4 mg/dL) and triglyceride (36.2 mg/dL) levels compared to patients who had never used COC. Ongoing oral contraceptive users also exhibited a 19.6 mg/dL greater rise in triglyceride levels relative to never-users, while no significant differences were observed in HDL cholesterol changes between groups [76]. Changes in lipid profiles were also dependent on the type of progestagen used in COCs. Formulations containing gestodene were associated with a significantly smaller increase in total cholesterol and triglycerides compared to those containing norgestimate or desogestrel, which may be attributed to gestodene’s lower androgenic activity [77]. The impact of reduced doses of estrogens on the lipid profile was evaluated. No significant differences were observed between the ethinyl estradiol doses of 20 μg and 30 μg composed with 100 μg and 150 μg of levonorgestrel, respectively [78].

The general use of oral contraception was analyzed in the context of HF development. Since the prevalence of heart failure rises with age, there are limited data on the association between contraceptive use in premenopausal women and the development of heart failure [79]. A recent cohort study of 3594 individuals using multivariable regression and inverse probability of treatment weighting models did not reveal neither an increased risk of HF development (HR 0.96; 95% CI 0.63–1.48; *p* = 0.86) nor left ventricular ejection fraction (LVEF) reduction. No effect of therapy duration was observed. However, the analysis revealed an increased risk of left ventricular end-diastolic mass and stroke volume [80]. Hormonal contraception was not related to HF exacerbation, but a tendency to fluid retention was notable [81].

### 11.2. Progestogens and Cardiovascular Risk

Unlike COCs, progestogen-only methods are thought to exert a different and potentially lower impact on cardiovascular risk. Progestogen-only contraception (POC) may offer a safer alternative for particular high-risk patients. A meta-analysis by Glisic M et al. showed no impact of oral and intrauterine POC formulations on VTE incidence. However, injectable POCs increased the risk by over 2–2.5 times compared with nonuse [81,82]. This result may be related to high serum progestogen levels after depot formulation administration and dose–response for VTE risk [83,84].

The influence of progestogens on arterial thrombosis has also been assessed, with risk expressed in terms of myocardial infarction and ischemic stroke incidence.

Fully adjusted models of the aforementioned meta-analysis revealed that POCs were not associated with an increased risk of MI incidence (RR 0,98, 95% CI 0.66–1.47) [85]. However, a large prospective Danish cohort study including over two million individuals demonstrated that current use of oral POCs was associated with a 1.5-fold higher risk of myocardial infarction compared with nonuse (RR 1.5; 95% CI, 1.1–2.1), but intrauterine levonorgestrel devices (LG-IUDs) did not show such a relationship. This study also presented an increased risk of ischemic stroke among POC pill patients in comparison to nonuse (RR 1.6; 95% CI 1.3–2.0). However, the study should be interpreted in the context of its serious limitation—a small number of discussed endpoints despite the large analyzed cohort [86].

Although the studies presented in the previously mentioned meta-analysis demonstrated diversified risk ratios considering the relationship between POC use and ischemic stroke, none of the results showed statistical significance [85]. Considering the formulations, neither implants nor pills increased the risk of stroke in the general population [82]. What is more, LG-IUD use assessed in the Danish study (2004–2021) was associated with even a decreased risk of ischemic stroke (RR 0.78; 95% CI 0.70–0.88) and did not elevate a risk of intracerebral hemorrhage (RR 0.94; 95% CI 0.69–1.28) [87]. Under high-risk clinical conditions, such as arterial hypertension, the relative risk of stroke demonstrated a substantial increase. Compared with nonusers of hormonal contraceptives without a history of hypertension, the odds ratio for stroke rose from 7.2 (95% CI 6.1–8.5) among nonusers to 12.4 (95% CI 4.1–37.6) among current users of POCs [88].

POCs did not demonstrate any effect on blood pressure elevation [85,89]. Moreover, drospirenone pills were associated with reductions in both systolic and diastolic blood pressure among women under 35 years of age with baseline values exceeding 130/85 mmHg [90]. Moreover, the drospirenone–progestagene component in COC was also associated with blood pressure decrease compared to levonorgestrel with ethinyl estradiol, which was related to drospirenone’s antimineralocorticoid activity with a natriuretic and diuretic effect [91].

There are limited data on the effects of POC on the lipid profile. A small prospective study demonstrated no influence on LDL cholesterol levels and a slight decrease in total cholesterol, HDL cholesterol, and triglyceride serum levels. The results were similar for equivalent doses of levonorgestrel and desogestrel [92]. A large cohort study by Wang Q. et al. showed that POC use did not exert any negative impact on the lipid profile, in contrast to COCs [93]. Although most studies have not demonstrated an adverse effect on lipid metabolism, a recent small study on women diagnosed with endometriosis treated with 2 mg of dienogest daily reported a statistically significant increase in serum triglyceride levels after a six-month observation period among primarily healthy young women [94].

### 11.3. Hormonal Contraception Summary and Cardiovascular Risk Considerations

To summarize, hormonal contraception remains a cornerstone of preconception planning and is particularly important in women diagnosed with cardiovascular disease. The effectiveness of contraceptive methods is categorized into three tiers. Tier I methods (with 1-year failure rates of <1%) include IUDs and implants, whereas Tier II methods comprise COCs and POCs, such as pills and injectable depot formulations, which have 1-year failure rates of 6–12% [95].

The 2016 and 2024 U.S. Medical Eligibility Criteria for Contraceptive Use (U.S. MEC) categorize medical conditions according to the recommendation of particular contraceptive method use [96,97].

According to the guidelines of the American College of Obstetricians and Gynecologists (ACOG) for women with congenital heart diseases, POCs are recommended as the first-line contraceptive option for women with valvular heart disease, regardless of the presence of complications. IUDs are a preferred option for individuals with a high risk of cardiac conditions due to their high efficiency profile. Furthermore, the reduction in menstrual bleeding represents an additional beneficial action, particularly in patients receiving anticoagulant therapy, as it lowers the risk of anemia. It may also be advantageous in women with postural tachycardia syndrome by helping to alleviate symptoms [98]. However, IUD use is not recommended in individuals at a high risk of infective endocarditis or in those with pulmonary hypertension due to the potential risk of a vasovagal reaction during cervical manipulation at the time of insertion [99].

According to the MEC guidelines, the use of IUDs represents a safe contraceptive option for patients with cardiovascular risk factors, such as older age, smoking, diabetes, hypertension, dyslipidemia, and obesity, as well as for those with acquired valvular heart disease, a history of peripartum cardiomyopathy, deep vein thrombosis, or PE. This recommendation applies regardless of the presence of active cancer, thrombophilia, or recent surgery (MEC category 1 or 2). IUDs and subcutaneous progestagen implants are also considered safe contraceptive options for patients who have overcome MI [100].

In contrast, the use of COCs is generally contraindicated in individuals with a history of deep VTE or PE (MEC category 3), especially in those at high risk of thrombosis, such as patients who have experienced thrombotic events despite receiving anticoagulants or those with thrombophilia (MEC category 4). In cases of uncomplicated valvular heart disease, the benefits of COCs typically surpass the potential or established risks (MEC category 2). However, the coexistence of some severe conditions, such as pulmonary hypertension, a risk for atrial fibrillation, or a history of subacute bacterial endocarditis, constitutes a contraindication to this form of treatment (MEC category 4). Peripartum cardiomyopathy also represents a cardiologic contraindication to COC use [97]. An overview of key studies evaluating the effects of hormonal contraception use on cardiovascular risk factors and cardiovascular events in women is presented in Table 4.

## 12. Conclusions

It remains disputable whether a low testosterone level is clearly related to higher cardiovascular and overall mortality or rather represents a surrogate marker of general health, including comorbidities and lifestyle. It is especially vital to stress the fact that diseases such as diabetes, obesity, and hypertension are acknowledged CV risk factors, parallelly being associated with male hypogonadism. This creates a sophisticated, bidirectional relationship between a low testosterone level and CV health, necessitating further investigation. TRT seems to be a safe therapeutic option, and benign prostatic hypertrophy, widely prevalent in elderly men, does not belong to the contraindications for TRT. In patients on TRT, the prostate size can increase, but it is no different from that of men without TRT. TRT may improve quality of life in hypogonadal men and show a positive influence on the CV system, potentially lowering or having a neutral effect on CV risk and mortality, representing a promising therapeutic option in patients at risk of HF, frailty, and metabolic syndrome [101,102]. Still, it is not free from controversies, with the results of past studies being influenced by the source of funding. The optimal indications for TRT are debatable, especially in males planning to reproduce, as TRT may affect fertility.

The initiation of TRT in elderly patients and patients with HF or at risk of HF should be approached with caution, utilizing a shared decision-making model that weighs the individualized risk–benefit ratio.

Similarly, in women, hormone replacement therapy (HRT) offers relief from menopausal symptoms and supports bone density, though its cardiovascular impact is multifaceted and depends on factors such as the type of hormones used, timing of therapy initiation, and route of administration. HRT is considered safe for low-risk women under 60 years, within 10 years of menopause onset, and without high CVD or breast cancer risk.

## Figures and Tables

**Table 1:** Recommendations for TRT based on age and testosterone concentration [4,13].

Age	Indication
40–49	TRT is recommended when TT < 300 ng/dL (10.4 nmol/L) with co-existing hypogonadal symptoms
50–80	when TT < 15 nmol/L with hypogonadal symptoms—TRT may be beneficial (fT < 225 pmol/L (65 pg/mL) or 243 pmol/L (70 pg/mL) support TRT administration)
	when TT < 12.1 nmol/L—indication to start TRT

fT—free testosterone, TRT—testosterone replacement therapy, TT—total testosterone concentration.

**Table 2:** Summarization of the major studies on TRT, CV risk, and mortality. ACS—acute coronary syndrome, CABG—coronary artery bypass grafting, CVD—cardiovascular disease, CV—cardiovascular, DVT—deep vein thrombosis, HF—heart failure, MI—myocardial infarction, MACCE—major adverse cardiac and cerebrovascular event, PCI—percutaneous coronary intervention, PE—pulmonary embolism, RCT—randomized controlled trial, T—testosterone, TRT—testosterone replacement therapy, UA—unstable angina.

Study	Study Design	Endpoint	Result	Limitations	Guideline Inconsistencies
Lincoff et al., 2023 [16] Cardiovascular Safety of Testosterone-Replacement Therapy	A multicenter, randomized, double-blind, placebo-controlled, noninferiority trial; 5246 men randomly assigned transdermal 1.62% testosterone gel or placebo gel	Occurrence of any component of a composite of death from CV causes, nonfatal myocardial infarction, or nonfatal stroke	TRT was noninferior to the placebo with respect to the incidence of MACCE	High rate of treatment discontinuations; short treatment and follow-up time	Heightened risk of MI/stroke during TRT
Wallis et al., 2016 [5] Survival and cardiovascular events in men treated with testosterone replacement therapy: an intention-to-treat observational cohort study	A population-based matched cohort study; 10,311 men treated with TRT and 28,029 controls	Mortality, CV events, and prostate cancer	Long-term TRT—reduced mortality, cardiovascular events, and prostate cancer; short-term TRT—higher mortality and cardiovascular events	Lack of randomization; possible selection and detection bias; no baseline T level	Intensive cardiac monitoring during the first 3–4 months of TRT was not suggested; TRT requires frequent prostate cancer screening
Baillargeon et al., 2014 [17] Risk of Myocardial Infarction in Older Men Receiving Testosterone Therapy	A population-based cohort; 6355 patients on TRT and 19,065 testosterone nonusers	MI	TRT was not associated with a heightened risk of MI; TRT seemed to be protective against MI in men from the highest-risk group	No assessment of actual T level; only intramuscular route of T administration	TRT should be avoided or used with extreme caution in men with high CV risk
Basaria et al., 2010 [18] Adverse events associated with testosterone administration	A randomized, controlled trial; 209 participants randomly assigned to receive placebo gel or testosterone gel	Primary endpoint—mobility improvement measured by leg-press strength; safety endpoints: MI, stroke, CV mortality, hospitalization for HF, UA, arrhythmias, and HT-related events	The application of a testosterone gel was associated with an increased risk of cardiovascular adverse events; TRT improved leg and chest-press strength	Study conducted on an extremely high-risk population; terminated early; may lack statistical power	Contemporary guidelines suggested TRT was safe if patients were screened for prostate cancer
Xu et al., 2013 [19] Testosterone therapy and cardiovascular events among men: a systematic review and meta-analysis of placebo-controlled randomized trials	A systematic review and meta-analysis of placebo-controlled randomized trials of TRT lasting 12+ weeks; encompassed 2994 men	CV events: MI, stoke, CV mortality, PCI, CABG, investigator-reported palpitations, peripheral edema, other HF symptoms, and new-onset arrhythmias	TRT increased the risk of cardiovascular-related events regardless of the baseline T level; the effect of TRT varied regarding the source of funding—in trials funded by the pharmaceutical industry, the CV risk was lower	Pooled small trials were inconsistent; broad endpoint, including minor events	Guidelines relying on pharmaceutical data might be incomplete in terms of safety
Corona et al., 2011 [1] Hypogonadism as a risk factor for cardiovascular mortality in men: a meta-analytic study	A meta-analysis of 6 RCT, 54 cross-sectional, and 10 longitudinal studies to evaluate the relationship between hypogonadism and CV mortality	Cross-sectional endpoint—prevalence of CV disease; longitudinal endpoint—all-cause mortality and CV mortality; RCT endpoint—changes in treadmill test parameters	Lower testosterone and higher 7-β estradiol levels were associated with increased risk of CVD and CV mortality	Included a small number of RCTs with 258 patients, and the follow-up was short (23 to 52 weeks); in the longitudinal and cross-sectional studies, there was an inability to determine if low T was the biomarker or the cause	It was a novelty for contemporary guidelines that a low T level is one of the CVD risk factors, as well as a high estrogen level
Corona et al., 2018 [20] Endogenous Testosterone Levels and Cardiovascular Risk: Meta-Analysis of Observational Studies	A meta-analysis of 37 observational studies, including 43,041 men	CV mortality and morbidity	Low T in aging men could represent a marker of CV risk	Many incorporated studies lacked complete follow-up and suffered from poor management of missing data; the link between low T and CV death was influenced by diabetes and active smoking	Guidelines recommend a strict T level threshold—in the study, men in the “low-normal” range still showed increased CV risk (linear association)
Shores et al., 2012 [6] Testosterone treatment and mortality in men with low testosterone levels	An observational study, encompassing 1 031 male veterans, testosterone-treated, compared with untreated men	All-cause mortality	In men with low T levels, testosterone TRT was associated with decreased mortality compared with no the TRT group	Lack of randomization; non-standardized testosterone level measurement; possible “Healthy User” selection bias	Contemporary guidelines focused on the potential risks of TRT, while according to this study, the real danger was not treating the patient with a very low T level
Gencer et al., 2021 [21] Prognostic value of total testosterone levels in patients with acute coronary syndromes	A prospective observational cohort study, including 1054 men hospitalized for ACS	All-cause mortality at one year	After adjustment for high-risk confounders, low T levels were not associated with mortality; a low T level was prevalent in almost 40%	T levels measured during acute cardiac event; one year follow-up; no interventional data	A low T level may serve as a mortality predictor, which is not included in guidelines; guidelines recommend testing T levels in stable and healthy patients
Sharma et al., 2016 [15] Association Between Testosterone Replacement Therapy and the Incidence of DVT and Pulmonary Embolism: A Retrospective Cohort Study of the Veterans Administration Database.	A retrospective study; 10,854 subjects without TRT and 60,553 with TRT (after treatment, 38,362 normal T, 22,191 low TT)	The incidence of DVT and PE during TRT therapy	TRT in patients with low–moderate risk of DVT/PE was not associated with an increased risk of DVT/PE	Recent surgery and immobilization not taken into consideration; excluded patients with prior DVT/PE/hypercoagulable state	Guidelines treat the history of VTE as a relative contraindication for TRT

**Table 3:** An overview of key studies evaluating the effects of HRT on cardiovascular risk factors and cardiovascular events in postmenopausal women.

Study	Study Design	Population Characteristics	Main Outcomes
Wassertheil-Smoller et al., 2003 [34]	Randomized controlled trial (WHI)	Postmenopausal women aged 50–79 years; conjugated equine estrogen (CEE) 0.625 mg/day + medroxyprogesterone acetate (MPA) 2.5 mg/day	Combined HRT associated with increased risk of ischemic stroke; no protective effect on cerebrovascular outcomes
Manson et al., 2003 [35]	Randomized controlled trial (WHI)	Women aged 50–79 years without prior CHD; CEE 0.625 mg/day + MPA 2.5 mg/day	No reduction in coronary heart disease risk; early increase in CHD events observed after initiation of therapy
Anderson et al., 2004 [36]	Randomized controlled trial (WHI)	Postmenopausal women aged 50–79 years with hysterectomy; CEE 0.625 mg/day	Estrogen-only therapy did not reduce coronary heart disease risk; increased risk of stroke observed
Yang et al., 2013 [37]	Meta-analysis of randomized controlled trials	Postmenopausal women receiving mainly standard doses (CEE 0.625 mg/day ± MPA 2.5 mg/day)	HRT did not reduce cardiovascular mortality or coronary events; increased risk of stroke and venous thromboembolism reported
Rossouw et al., 2007 [40]	Secondary analysis of randomized controlled trials (WHI)	Women aged 50–79 years; stratified by <10, 10–19, ≥20 years since menopause	CVD risk varied by age and years since menopause; younger women and those closer to menopause showed lower absolute CVD risk, but no overall cardiovascular benefit; increased risk of stroke and venous thromboembolism
Boardman et al., 2015 [41]	Cochrane systematic review and meta-analysis	Postmenopausal women receiving hormone therapy (oral and transdermal) for primary or secondary CVD prevention	Hormone therapy did not prevent cardiovascular disease; increased risk of stroke and venous thromboembolism; evidence does not support HRT for CVD prevention
Hodis et al., 2016 [43]	Randomized controlled trial (ELITE)	Healthy women aged 55–79 years; early (<6 years) vs. late (≥10 years) postmenopause; oral estradiol 1 mg/day (or transdermal 50 µg/day) with vaginal progesterone in women with a uterus	Early initiation of estradiol slowed progression of subclinical atherosclerosis; no benefit observed with late initiation, supporting the timing hypothesis
Bergendal et al., 2016 [44]	Population-based case–control study	Peri- and postmenopausal women using local or systemic hormone therapy	Systemic oral estrogen associated with increased VTE risk; transdermal and local estrogen showed lower or no increased VTE risk
Goldštajn et al., 2023 [45]	Systematic review	Postmenopausal women; comparison of oral vs. transdermal estrogen at standard therapeutic doses	Transdermal HRT associated with more favorable cardiovascular and thrombotic risk profile compared with oral therapy
Straczek et al., 2005 [46]	Case–control study	Postmenopausal women, including carriers of prothrombotic mutations; oral vs. transdermal estrogen	Oral estrogen markedly increased VTE risk, especially in women with thrombophilic mutations; transdermal estrogen showed no significant risk increase
Wakatsuki et al., 2002 [47]	Randomized controlled trial	Postmenopausal women; oral CEE vs. transdermal estradiol (standard doses)	Oral estrogen increased LDL particle size but also oxidative susceptibility; transdermal estrogen had more neutral metabolic effects
Cushman et al., 2004 [49]	Randomized controlled trial (WHI)	Women aged 50–79 years; CEE 0.625 mg/day + MPA 2.5 mg/day	Combined HRT significantly increased risk of venous thrombosis, particularly during the first year of therapy
Shimbo et al., 2014 [50]	Randomized controlled trial (WHI)	Postmenopausal women 50–79 years, mean 12–13 years since menopause; WHI participants without prior CVD; CEE 0.625 mg/day ± MPA 2.5 mg/day; BP measured longitudinally	Hormone therapy slightly increased mean blood pressure and blood pressure variability, with potential implications for cardiovascular risk
de Lauzon-Guillain et al., 2009 [52]	Prospective cohort study	Postmenopausal women using predominantly transdermal HRT	HRT associated with reduced risk of new-onset diabetes, particularly with transdermal estrogen

BP—blood pressure; CEE—conjugated equine estrogen; CHD—coronary heart disease; CVD—cardiovascular disease; HRT—hormone replacement therapy; LDL—low-density lipoprotein; MPA—medroxyprogesterone acetate; VTE—venous thromboembolism; WHI—Women’s Health Initiative.

**Table 4:** An overview of key studies evaluating the effects of hormonal contraception use on cardiovascular risk factors and cardiovascular events in women.

Study	Study Design	Population Characteristics	Main Outcomes
Zhao et al., 2018 [59]	Prospective cohort	Postmenopausal women without baseline cardiovascular disease, predominantly aged ≥ 60 years, recruited from a large US community-based cohort	Higher endogenous estradiol associated with increased CVD risk
Bhullar et al., 2024 [60]	Narrative review	Women of reproductive age using combined and progestin-only oral contraceptives	Increased risk of VTE, hypertension, MI, and stroke with combined OCs
Skafar et al., 1997 [61]	Clinical review	Women across the lifespan, including premenopausal, perimenopausal, and postmenopausal age groups	Estrogen protective; progestins show heterogeneous CV effects
Thomas and Pang, 2013 [62]	Experimental/review	Animal models and adult human cardiovascular cell lines	Progesterone shows rapid protective effects
Sidney et al., 2004 [64]	Case–control	Premenopausal women, most aged 18–44 years	Low-estrogen combined oral contraceptives were associated with higher risk of venous thromboembolism compared with nonusers
Lidegaard et al., 2009 [65]	Nationwide cohort	Danish women aged 15–49 years without prior thromboembolism	Hormonal contraception, particularly combined oral contraceptives with higher estrogen doses and certain progestin types, was associated with a significantly increased risk of venous thromboembolism in women aged 15–49, though absolute risk remained low
Oedingen et al., 2018 [66]	Systematic review and meta-analysis	Women of reproductive age using COCs (typically 15–49 years)	Higher VTE risk with newer progestins and higher EE doses
Cameron et al., 2023 [67]	Narrative review	Women of reproductive age and early perimenopause	Oral contraceptives associated with modest BP increases
Park and Kim, 2013 [68]	Cross-sectional	Korean women aged 20–49 years	OC use associated with hypertension and prehypertension
Weir, 1982 [69]	Interventional	Adult women, mainly 20–40 years, with OC-induced hypertension	BP reduction after switching to low-dose formulations
Roach et al., 2015 [70]	Systematic review (Cochrane)	Premenopausal women, mostly <50 years	Increased risk of MI and ischemic stroke
Rosenberg et al., 1980 [71]	Case–control	Premenopausal women aged approximately 20–44 years	OC use increases nonfatal MI risk
Stampfer et al., 1988 [72]	Prospective cohort	Healthy women, baseline age mainly 30–55 years	No increased long-term CVD risk after past OC use
Dou et al., 2023 [73]	Prospective cohort	Middle-aged women, mean age ~55–60 years (UK Biobank)	OC use associated with lower all-cause mortality
Fan et al., 2024 [74]	Prospective cohort	Women of reproductive age, generally 20–49 years	Reproductive factors (parity, age at menarche/menopause) and hormonal contraceptive use were associated with differential risk of type 2 diabetes, with genetic susceptibility further modifying this risk; certain combinations of reproductive history and genetic profile significantly increased diabetes incidence
Snell-Bergeon et al., 2008 [75]	Observational cohort	Women with type 1 diabetes, mean age ~40 years	Hormonal contraception linked to coronary calcium progression
Staudt et al., 2024 [76]	Longitudinal cohort	Healthy adolescent girls aged ~14–19 years	Adolescent OC users exhibited changes in lipid metabolism, suggesting early monitoring of lipid profiles may be warranted during hormonal contraceptive use
Skouby et al., 2005 [78]	Randomized controlled trial	Healthy women aged ~18–40 years	Lower EE dose improved lipid and glucose metabolism
Luo et al., 2021 [80]	Prospective cohort	Adult women, mainly 40–60 years at baseline	OC use associated with incident heart failure
Stachenfeld and Keefe, 2002 [81]	Experimental	Healthy adult women	Estrogen influenced AVP secretion and fluid balance
Tepper et al., 2025 [82]	Systematic review	Women of reproductive age using progestin-only contraception	No significant increase in thrombosis risk
Cockrum et al., 2022 [84]	Case–control	Women aged 18–49 years	VTE risk differed by progestogen type; higher for desogestrel and drospirenone compared with levonorgestrel
Glisic et al., 2018 [85]	Systematic review and meta-analysis	Women of reproductive age using POPs	POP use not associated with adverse cardiometabolic outcomes; generally neutral effects on BP, lipids, and glucose
Yonis et al., 2025 [86]	Nationwide prospective cohort	Women aged 15–49 years	Hormonal contraception slightly increased risk of stroke and MI; absolute risk low, influenced by age, smoking, and hormone type
Letnar et al., 2024 [87]	Cohort study	Women of reproductive age using LNG-IUD	No increased ischemic stroke risk
WHO Collaborative Study, 1998 [88]	Multicenter case–control	Women aged 15–49 years from multiple countries	Minimal CVD risk with progestogen-only methods
Hussain, 2004 [89]	Literature review	Women of reproductive age using POPs	No consistent BP elevation
Kimble et al., 2020 [90]	Prospective phase 3 trial	Healthy women aged 18–45 years	Favorable safety and BP profile
Oelkers et al., 1995 [91]	Interventional study	Adult women of reproductive age	Drospirenone OC lowered renin–aldosterone activity, modestly reduced BP, and maintained neutral/favorable metabolic profile
Barkfeldt et al., 2001 [92]	Comparative trial	Women aged ~18–45 years	Desogestrel POP caused slightly higher total cholesterol and LDL increases than levonorgestrel; HDL and triglycerides largely unchanged
Wang et al., 2016 [93]	Cross-sectional and longitudinal	Population-based cohort, mostly <50 years	HC use altered systemic metabolism, affecting lipids and amino acids; effects dependent on contraceptive type and duration

BP—blood pressure; COC—combined oral contraceptive; CVD—cardiovascular disease; EE—ethinyl estradiol; HDL—high-density lipoprotein cholesterol; LNG-IUD—levonorgestrel-releasing intrauterine device; LDL—low-density lipoprotein cholesterol; MI—myocardial infarction; OC—oral contraceptive; POP—progestin-only pill; VTE—venous thromboembolism.

## Data Availability

Data are contained within this article.

## Abbreviations

The following abbreviations are used in this manuscript:

CVD	cardiovascular disease
HRT	hormone replacement therapy
TRT	testosterone replacement therapy
CV	cardiovascular
HF	heart failure
VTE	venous thromboembolism
LOH	late-onset hypogonadism
HpEF	heart failure with preserved ejection fraction
HIV	human immunodeficiency virus
EAU	European Association of Urology
SHBG	sex hormone-binding globulin
CAD	coronary artery disease
TT	total testosterone concentration
fT	free testosterone
MI	myocardial infarction
HDL	high-density lipoprotein
MACE	major adverse cardiovascular event
TOM	The Testosterone in Older Men with Mobility Limitations
DHEA	dehydroepiandrosterone
TC	total cholesterol
LDL	Low-density lipoprotein
TG	triglyceride
HbA1c	hemoglobin A1c
FPG	fasting plasma glucose
BP	blood pressure
6MWT	6-minute walk test
peak VO2	peak oxygen consumption
NYHA	New York Heart Association
AAS	anabolic androgenic steroid
LV	left ventricle
RAAS	renin–angiotensin–aldosterone system
EF	ejection fraction
RV	right ventricle
CCTA	coronary computed tomography angiography
FSH	follicle-stimulating hormone
LH	luteinizing hormone
DM	diabetes
WHI	Woman’s Health Initiative
USA	the United States
HR	hazard ratio
CI	confidence interval
NO	nitric oxide
ELITE	Early Versus Late Intervention Trial With Estradiol
CIMT	carotid artery intima-media thickness
ASCVD	atherosclerotic cardiovascular disease
TIA	transient ischemic attack
PE	pulmonary embolism
GAHT	gender-affirming hormone therapy
OR	odds ratio
SMR	standardized mortality ratio
COC	combined oral contraception
RR	relative risk
T2DM	type 2 diabetes
T1DM	type 1 diabetes
LVEF	left ventricular ejection fraction
POC	progestogen-only contraception
LG-IUDs	intrauterine levonorgestrel devices
IUDs	intrauterine devices
U.S. MEC	U.S. Medical Eligibility Criteria for Contraceptive Use
ACOG	American College of Obstetricians and Gynecologists
